# Qualitative enquiry on factors affecting antibiotic prescribing at primary healthcare facilities in Addis Ababa, Ethiopia

**DOI:** 10.3389/fmed.2024.1308699

**Published:** 2024-04-08

**Authors:** Fikru Worku Altaye, Gloria Thupayagale-Tshweneagae, Faniswa Honest Mfidi

**Affiliations:** ^1^United Nations Population Fund, Addis Ababa, Ethiopia; ^2^Centre for Health Research and Community Development, Gaborone, Botswana; ^3^Department of Health Studies, University of South Africa, Pretoria, South Africa

**Keywords:** antibiotics, prescribing practice, in-depth interview, factors, key informants, primary health care, prescribers

## Abstract

**Background:**

The major driver of antibiotic resistance is the huge increase in antibiotic prescribing, especially in low- and middle-income countries.

**Aim:**

This study aimed to explore factors affecting antibiotic prescribing at primary healthcare facilities in Addis Ababa, Ethiopia.

**Methods:**

The study was part of a three-phased explanatory sequential mixed method design. The study was conducted in five primary healthcare facilities through in-depth interviews of 20 prescribers and 22 key informants using pre-prepared interview guides. The data were analysed through thematic content analysis by applying ATLAS.ti 9 software.

**Results:**

The antibiotic prescribing decision of prescribers was shown to be affected by various factors. The factors related to prescribers include not updating oneself on antibiotic use and antibiotic resistance, not reviewing patient history, not considering the concerns related to antibiotic resistance during prescribing, and competency problems. The patient-related factors were low awareness about antibiotics, lack of respect for prescribers, and pressure on prescribers. The shortage of antibiotics and laboratory reagents, a lack of updated antimicrobial resistance information, patient load, inadequate capacity, private sector practice, inadequate follow-up and support, and health insurance membership were the system-related factors. Appropriate interventions should be designed and implemented to address the identified factors and improve the prescribing practice.

## 1 Introduction

Antimicrobial resistance (AMR), especially resistance to antibiotics, has become a worldwide challenge to public health (1). Patients infected with resistant organisms have an increased risk of poor clinical outcomes, including death, and consume more healthcare resources. Antibiotics are unique because they are the only pharmaceutical agents that have transmissible loss of efficacy over time (2). Currently, 700,000 people die each year worldwide from drug-resistant infections, and if there is no effective international action, scenario analysis has indicated that by the year 2050, this will increase to 10 million deaths annually (3). If AMR is left unchecked, by 2050, the world will be producing between 2 and 3.5% less than it otherwise would (4), pushing 28 million people into extreme poverty (5).

Since the major driver of antibiotic resistance is the huge increase in antibiotic prescribing, especially in low- and middle-income countries (LMICs) (6), the healthcare providers’ prescribing behaviour is an important area to promote the rational use of antibiotics (7). Although it is a global concern, inappropriate use of antibiotics and the resulting consequences of antibiotic resistance are greater in LMICs (8). Primary care is responsible for approximately 80% of the antibiotics consumed worldwide (9, 10). Evidence shows that most studies investigating the magnitude and determinants of antibiotic use have focused on high-income countries, and those from LMICs have been carried out predominantly in hospital settings (11). This highlights the need to focus the research and action at this level of healthcare (12). This study was conducted in selected public primary healthcare facilities found in Addis Ababa city, the capital city of Ethiopia. Studies conducted on the rate of antibiotic resistance in Ethiopia have shown that most of the bacteria that cause infections have developed a considerable degree of resistance to commonly used first-line antibiotics. The resistance pattern in the city administration where this study was conducted is also alarming (13–15).

To the best of the authors’ knowledge, there are no published studies conducted at primary healthcare facilities aimed at exploring the factors influencing antibiotic prescribing and designing interventions to improve antibiotic prescribing in the country in general and in Addis Ababa, in particular. This study was conducted to identify factors affecting the prescribing of antibiotics at primary healthcare facilities in the Addis Ababa city administration.

## 2 Materials and methods

### 2.1 Study design

This study reports on the qualitative component of an explanatory sequential mixed-method design conducted through in-depth interviews.

### 2.2 Setting

The study was conducted in Addis Ababa city, the capital city of Ethiopia. Administratively, the city is divided into 10 sub-cities and 121 districts and is estimated to have a total population of 3,854,866 (1,819,241 male and 2,035,625 female population) according to the population projection for 2022 of the 2007 National Population and Housing Census (16). In the public sector, health centres are the health facilities that provide primary healthcare in the city.

### 2.3 Study population and sampling strategy

The study population consisted of healthcare providers who were involved in patient diagnosis and prescribing medicines in the selected public health centres during the data collection period and office holders in the health centres, sub-cities, and the Health Bureau. The healthcare providers included in the study fulfilled the following criteria:

- Working in the selected healthcare facilities involved in patient diagnosis and prescribing of medicines for the past 2 years or more;
- Those who were on duty during the data collection period;
- Those who were willing to take part in the study; and
- Office holders in the selected health centres, sub-cities, and the Health Bureau.

Based on the preliminary findings of the quantitative phase of the study, one health centre was selected from each of the five sub-cities using the rate of antibiotic prescribing as a general selection criterion. The 10 health centres in which the quantitative study was conducted were divided into three groups: high, medium, and low rates of antibiotic prescribing. Then, one health centre was randomly picked from each group until five health centres were selected without repeating a health centre from a specific sub-city.

For the purposeful qualitative sample reported here, the sample selection was guided by criterion sampling (17). From each health centre, four prescribers who showed interest in participating in the study were selected for the in-depth interview. Given the limited number of prescribers at the health centre level, the decision was made to select four prescribers from each health centre representing the different clinical units (adult outpatient, paediatrics outpatient, and emergency units). This made the total number of prescribers included in the study to be 20. Accordingly, four prescribers who showed interest in participating in the study were purposively selected for the in-depth interview. Consideration was given to having prescribers from different clinical units (adult outpatient, paediatrics outpatient, and emergency units). Qualification and gender were also considered when selecting prescribers for the study. In the key informant interview, medical directors and pharmacy case team coordinators of the five health centres, coordinators of the medical service and pharmacy service and logistics teams of health offices of the five sub-cities, and Directors of the Medical Service and Pharmacy Service & Logistics Directorates of the City Administration Health Bureau were included in the study because of their service in healthcare and logistics managerial role at each level. Thus, the selection of the 22 key informants from the 11 offices (2 from each) was automatic.

### 2.4 Data collection

Data were collected from 1 May to 10 June 2021 by the first author and a trained research assistant. Three in-depth interview guides were used for interviewing prescribers, key informants from health centres, and key informants from sub-city health offices and Health Bureau. The guides were developed based on the objectives of the study and phases of the theoretical model used and were updated by preliminary findings of the quantitative study. Although most of the questions included in the guides were similar, there were questions specifically addressed to prescribers and key informants, given their role in the prescribing practice. The interview guides were first developed in English language and then translated into Amharic language. The guides were checked for loss of meaning by the first author and the research assistant, who is conversant in both languages, and re-checked by the second author, who is an expert in qualitative studies.

Before the actual data collection, two of the data collection guides (the guide for prescribers and the guide for key informant interviews at health centres) were pre-tested in two public health centres that were randomly picked from two of the sub-cities selected for the study, and some modifications were made on the probing questions. The guide used for the in-depth interview with prescribers contained six main and a series of probing questions focusing on the use of antibiotics, commonly used antibiotics and their indications, antibiotic resistance, problems associated with the prescribing and use of antibiotics, factors influencing antibiotic prescribing, interventions implemented so far to improve antibiotic prescribing, interventions suggested to be implemented to improve the prescribing of antibiotics in the study setting, and challenges that might be faced in implementing the suggested interventions to improve antibiotic prescribing.

Six main and a series of probing questions were included in the guide used for the key informant interview at health centres. The questions were designed to understand the views of the key informants on the use of antibiotics and the concerns related to antibiotic resistance in general, prescribing of antibiotics at health centres, factors influencing antibiotic prescribing, interventions implemented so far, interventions that should be implemented, and anticipated challenges in implementing the suggested interventions. For the key informants from sub-cities and the Health Bureau, the interview guide included questions in relation to the inclusion of antibiotic and antibiotic resistance-related issues in the annual work plans, performance reports, and supportive supervision activities, factors influencing antibiotics prescribing, interventions implemented so far, interventions that should be implemented to improve the prescribing of antibiotics at health centres, and implementation challenges. This manuscript presents the findings related to the factors influencing antibiotic prescribing.

The interview with both prescribers and key informants was conducted in Amharic language. Unique identifiers that contained the interviewee profession (NU – Nurse and HO – Health Officer) for prescribers and the role (PS – Pharmacy Service and MS – Medical Service) of key informants plus a two-digit number that shows the order of the interview for each category was used for each of the study participants. For instance, HO03 is the 3rd health officer interviewed in the prescribers’ category and MS08 is the 8th Medical Service manager/team lead interviewed in the key informants’ category. In each of the selected health centres, prescribers from different clinical units that fulfill the inclusion criteria were approached by the researcher and research assistant. For prescribers willing to participate in the study, a briefing on the objectives of the study, the data to be collected, the method of data collection, and the confidentiality of the information they would be providing was provided at their workplace. Most of the interviews were conducted on the day of the visit to the health centre. An alternate date and time were agreed upon with those prescribers who were not able to make the interview on the initial date of the visit.

Before starting the one-on-one interviews, permission was requested for audio recording, and each participant signed the consent form after reading the information sheet. Demographic information of the participants, such as age, qualification, position/role, years of experience, and service unit, was collected at the beginning of the interview. Although no new information emerged from the prescribers interviewed, starting from the 17th prescriber (information saturation), all 20 prescribers were interviewed.

### 2.5 Data analysis

The data collected from the prescribers and key informants was first transcribed verbatim by the researcher and research assistant from the audio record to text form in Amharic language. Each transcript was then translated into English by the researcher and randomly checked by the second author for consistency. The Amharic and English translations of the transcripts were sent back to all of the study participants via telegram for member checking. Study participants were communicated via telephone calls and short message service as well as in person to get their feedback and confirmation that the transcribed texts were their own words. The editorial comments provided by a few study participants were addressed in the respective transcripts.

After reading each transcript at least twice to establish a general impression of the information contained in it, the transcripts were entered into ATLAS.ti 9 for coding and analysis. Two sets of projects were created on the ATLAS.ti 9 software; one for the data from prescribers and the other for data from key informants. Each transcript was entered as a word document identified by the unique identifier used for each prescriber and key informant, which was consistent with the data management and analysis approach described in the literature (18). The data collected from prescribers and key informants were analysed separately by applying the same procedure. The socio-demographic characteristics of the participants were analysed using Microsoft Excel.

Guided by the research questions and the main questions of the interview guides, a code book that contained the themes and sub-themes with definitions was developed. Supported by the software, each of the transcripts was then coded thematically into seven themes, which were later split into sub-themes and categories by going through the transcripts line-by-line as described by Graneheim and Lundman (19). The topic of this study, factors affecting the prescribing of antibiotics, was one of the themes that emerged from the thematic analysis. From each transcript, responses of the study participants that talk about factors were coded as “Factors” as a theme, and then specific types of factors were identified and coded as sub-themes. Relevant quotations from each of the identified factors are categorised as sub-themes to substantiate the presentation and discussion of the findings. It was to present the finding in a more systematic manner that the specific factors affecting antibiotics prescribing decisions are re-categorised into prescriber-related, patient-related, and system-related factors, as presented in Table 1. Abbreviations and acronyms used by the study participants are presented as they are in the quoted texts. Participants have used abbreviations, such as amoxa (to mean amoxicillin), and acronyms, such as DIS (to mean Drug Information Service). The outputs of the thematic analysis were reviewed by the second and third authors in reference to the transcripts to ensure data accuracy (see Table 2).

**Table 1:** Factors affecting antibiotic prescribing at primary healthcare facilities in Addis Ababa, May to June 2021.

Theme	Sub-theme	Category
Factors affecting antibiotic-prescribing decision	Prescriber-related factors	Not updating oneself on antibiotic use and resistance Competency problem Not taking patient history Not taking AMR into consideration Prescriber’s negligence
	Patient-related factors	Patient pressure Low community awareness Not respecting healthcare providers
	System-related factors	Impact of the private sector CBHI membership Availability of antibiotics Availability of laboratory tests Lack of updated AMR information Not engaging pharmacy professionals Inadequate capacity building Lack of accountability Inadequate support and follow-up Lack of guidelines Patient load

**Table 2:** Background information of the key informants, May to June 2021.

Variable	Number	Percent
*Gender*		
Female	13	59.1
Male	9	40.9
*Age (Years)*		
20–30	4	18.2
31–40	14	63.6
41–50	2	9.1
> 50	2	9.1
*Profession*		
Health Officer	2	9.1
Professional Nurse	4	18.2
Public Health	4	18.2
Pharmacist	11	50
Medical Laboratory	1	4.5
Technology	2	9.1
*Total work experience (Years)*		
5–10	15	68.2
10–20	4	18.2
> 20	3	13.6
*Responsibility*		
Health centre medical director	5	22.7
Health centre pharmacy team coordinator	5	22.7
Sub-city health office medical service team leader	5	22.7
Sub-city health office pharmaceuticals and medical equipment supply and distribution team leader	5	22.7
Health bureau medical service directorate director representative	1	4.5
Health bureau pharmaceutical supply and pharmacy service directorate director	1	4.5

### 2.6 Ethics considerations

Ethics clearance was obtained from the Research Ethics Review Committee of the College of Human Sciences, University of South Africa (UNISA) with CREC Reference Number 64093352_CHS_CREC_2020. Based on this clearance, ethics approval was obtained from the Research Ethical Clearance Committee of Addis Ababa City Administration Health Bureau. Permission to conduct the study was granted through official letters from the ethics committee to sub-city health offices and then from the sub-cities to the selected health centres. Informed consent was obtained in written form from each of the prescribers and key informants before data collection. Participation in this study was on a voluntary basis, and participants were informed that their participation was voluntary and that they had the right to discontinue their participation at any point during the interview if they wished. Confidentiality of the data and reports generated from it was maintained by using codes in place of names and other personal identifiers.

## 3 Results

### 3.1 Background information on the health centres

According to the 2012 EFY (July 2019 to June 2020) data of the Health Management Information System (HMIS) of the health centres, infectious diseases accounted for approximately 57.1% of the top 10 diseases during the budget year ranging from 23% in TBHC to 65% in WD9HC. URTIs, including tonsillitis, common cold, and unspecified upper respiratory tract infections (URTIs), were the first in the list, accounting for 29.3% of the top 10 diseases and 51.4% of the infectious diseases in the top 10 list.

### 3.2 Background information of the study participants

A total of 42 participants were involved in the in-depth interview: 20 prescribers and 22 key informants. Confirmation of the correctness of the transcripts taken from the audio recording of each interview was obtained from 19 (95%) of the prescribers and 21 (95.5%) of the key informants who participated in the study, with an overall confirmation rate of 95.2%. The participants confirmed that the information transcribed from the audio records was their own.

#### 3.2.1 Background information of the prescribers

A total of 20 prescribers (4 from each health cesntres) participated in the in-depth interview. Almost all of the prescribers reported that they had not participated in any training related to antibiotics and antibiotic resistance during the past 2 years preceding the study (Table 3). The average time taken to undertake each in-depth interview was 34.7 min.

**Table 3:** Background information of the prescribers, May 2021.

Variable	Number	Percent
*Clinical unit*		
Adult OPD	7	35
Under 5	7	35
Emergency	6	30
*Gender*		
Female	12	60
Male	8	10
*Age (Years)*		
20–30	10	50
31–40	6	30
41–50	3	15
> 50	1	5
*Profession*		
Health officer	10	50
Professional nurse	8	40
Clinical nurse	2	10

#### 3.2.2 Background information of the key informants

A total of 22 participants from the five health centres (10), sub-city health offices (10), and Health Bureau (2) were involved in the key informant interviews. The individual interviews took an average of 43 min.

### 3.3 Themes, sub-themes, and categories

*“*Factors affecting the prescribing of antibiotics” was one of the seven themes that emerged from the thematic content analysis of the qualitative data. This theme was further split into sub-themes and then into categories, as presented in Table 1. Each of the sub-themes and the categories under them are described below, supported by relevant quotes from prescribers and/or key informants.

#### 3.3.1 Sub-theme 1: prescriber—related factors

These are factors that arise from the healthcare providers that are involved in prescribing antibiotics.

##### 3.3.1.1 Not updating oneself on antibiotic use and resistance

One of the factors raised by key informants affecting antibiotic prescribing was in relation to the prescribers themselves, not keeping themselves updated on antibiotic use and resistance.

“What I see on professionals is not updating oneself timely…This is the gap on professionals, not updating oneself regularly in relation to medicines.” (MS08).

Some of the key informants related the problem of not updating oneself with the failure to use available information resources such as Drug Information Service (DIS) and not applying the feedback provided by pharmacy professionals.

“There is DIS, but, there is no one coming and requesting information. There is information here with us about antibiotics. But, most people do not use and there is information gap. This contributes for drug resistance.” (PS01)

##### 3.3.1.2 Competency problem

The competency of the professionals involved in the prescribing of medicines was the other factor affecting prescribing practices. Key informants reported that there are professionals who are not competent enough to manage patients and, hence, to prescribe medicines. Some of the key informants linked this competency problem with the educational system of the country, which is not appropriately preparing graduates for the intended practice, especially in the private sector.

MS11 related the failure to realise health facility-based skill laboratory for the observed competency problem. According to PS04, the competency issue comes from the decision on who should prescribe, given the educational preparation of the professionals. This key informant suggested that prescribers should be physicians or health officers.

##### 3.3.1.3 Not reviewing patient history

Both prescribers and key informants reported this malpractice as a contributing factor to the irrational prescribing of antibiotics.

“The first is that there is gap from the provider side to go back and review patient’s medical history. There is a problem to look at previous history and its impact on the current complaint.” (MS01)

Some participants reported that even if the provider wants to review the patient’s past medical history, it is also difficult to get patient history sheets.

“The second is loss of history sheets. History sheets are lost even in medical record room. Sometimes, medical records of patients that were in use for long time don’t have history sheets inside. Disorganization of the card room, lack of computerized recording system, and time constraint are also factors.” (NU10)

MS01 related the problem to patients who failed to reveal past medical history to healthcare providers.

##### 3.3.1.4 Failure to consider AMR

The issue of resistance not being considered during prescribing was another factor affecting antibiotic prescribing. When asked about their view on the practice of taking AMR into consideration during prescribing, MS01 responded

“I think there is gap on that. I think there is gap in thinking about the long-term implications beyond undertaking the daily routines. I consider that as a gap in the facility.”

This was supported by other key informants (MS02, PS01, and PS05). MS03 and PS04 indicated that prescribers take antibiotic resistance into consideration when prescribing.

##### 3.3.1.5 Prescriber’s negligence

The negligence of providers was mentioned by some prescribers as a factor in the prescribing of antibiotics.

“What I think as a problem is negligence. If a patient treated with a specific antibiotic shows no improvement, we should think one step up instead of down.” (HO08)

#### 3.3.2 Sub-theme 2: patient-related factors

Study participants reported that patients have their own contributions to the prescribing practice.

##### 3.3.2.1 Patient pressure

Patient pressure was the most commonly mentioned factor affecting the prescribing of antibiotics. Patients coming to health facilities want specific antibiotics during each health facility visit and push providers for that. They do that even for minor cases. Convincing patients to manage minor cases at home as per the guidelines is not easy as that has not been the practice in the past, according to the study participants.

“Patients want to have antibiotic for any flu case since they used to get that from private health facilities. I have personal observation in the private related with flu. For a case that you think requires hot drinks and home-based management, they prescribe ceftriaxone.” (HO04)

The prescribers indicated that the patient pressure is to the extent that healthcare providers prescribe antibiotics for the patient just to avoid conflict with the patient and subsequent grievance.

“Most patients will be very happy if you give them ceftriaxone injection instead of amoxicillin. I think most professionals are coming to say that “why should I quarrel with the patient?” and intend to prescribe as per the patient’s interest.” (NU05)

Some prescribers indicated that despite patient pressure, they do not change their prescribing decision.

“They complain when you tell them that medicine is not required. But, we don’t prescribe though they push us to do that.” (HO04)

##### 3.3.2.2 Low community awareness

The community’s awareness of antibiotics and resistance was another common factor mentioned by study participants.

“The community’s awareness is low. Patient’s focus is on getting fast relief and there is a problem of not thinking about the long-term complications related with the misuse.” (MS01)

One key informant raised the lack of public awareness of the implementation of the new guidelines, PHCG.

“As per the PHCG, there are cases that are sometimes managed without medication and requires only counselling. The patient is not familiar with this kind of management.” (PS02)

##### 3.3.2.3 Disregarding healthcare providers

One of the prescribers raised the ethical problem of patients affecting and influencing the prescribing practices.

*“The respect given to professionals in the past is different from the current one. Just imagine how you manage the next patient after being insulted by a patient and passed through unnecessary conflict. So, one is the ethical problem related with patients.” (HO03)*

#### 3.3.3 Sub-theme 3: system-related factors

Factors arising from the healthcare system that affects the prescribing practice are categorised under this sub-theme.

##### 3.3.3.1 CBHI membership

The CBHI membership status of patients was reported as a factor affecting the antibiotic prescribing decisions of prescribers. One prescriber (HO01) indicated that prescribers are very reluctant to prescribe medicines if the patient is a CBHI member due to fear of accountability.

“From the beginning, we ask clients “Are you CBHI or paying patient?” If the client is paying one, we are not afraid of writing medications. If CBHI, professionals send the patient mostly with no medication. This is because there is audit and recording on finance.” (HO01)

Other prescribers reported that patients who are members of the CBHI scheme want to be given medicines at every visit, considering it as their right in compensation for the premium they are paying for the insurance scheme. It is a form of patient pressure on providers backed by CBHI membership.

Key informants also raised concerns about the increase in the prescribing of antibiotics due to the CBHI scheme, in that patients visit health facilities more frequently and take medicines at every visit.

“In our health facilities, what is coming as a new problem related with medications is Community-Based Health Insurance. When prescriptions are audited and observed in various ways, we are identifying that unnecessary medications are being prescribed for clients. The number of patients receiving antibiotics is increasing and patients are visiting health facilities repeatedly due to the feeling they have that “I have paid” or “it is paid for me”.” (MS07)

##### 3.3.3.2 Lack of updated AMR information

Most of the key informants reported that there is a lack of updated information on the local antibiotic resistance situation. Although some of the key informants from the Health Bureau and sub-city health offices reported sharing various information via telegram, including about AMR, most participants indicated the lack of updated information on AMR. A key informant (MS11) indicated that the only opportunity he had to hear about antibiotic resistance was at a workshop organised by the Health Bureau. This was supported by other key informants (MS11 and PS01) who reported the difficulty in obtaining updated resistance information.

“We know about the problem from discussion. But, I haven’t seen any document that specifically shows the resistance situation in a specific facility or in the city supported by figure.” (MS11)

“There is gap in updating the staff with information. Once you are assigned here, you always do the same thing without getting any updated information.” (PS01)

##### 3.3.3.3 Inadequate capacity building

Problems associated with the capacity building of professionals on antibiotic use and resistance were the other factors commonly mentioned by the study participants. It is reported that training is not common in the area of medicine use in general and on antibiotics and resistance, in particular, as a country, although there is training in other areas. Prescribers were quoted saying the following in relation to capacity building on antibiotics and resistance:

“The things that can be causes are lack of refreshing the professionals on regular basis.” (HO10)

“Is proper training being given on curative aspect? As you see, there is no training on curative aspect. The focus is on disease prevention.” (HO03)

Key informants supported the claim from prescribers. MS01 indicated that there was no training for prescribers.

“To be honest, other than the one I mentioned before, I don’t think there was training, especially for prescribers who provide services in OPDs. I don’t remember any event other than that workshop.” (MS01)

According to MS04, training on antibiotic use is not common despite the presence of training in other areas.

“The other thing is that many training opportunities are given to capacitate the provider. But, training on antibiotics use and overall on medicines use are not common even as a country.” (MS04)

PS08 from the sub-city health office complained about the absence of capacity-building training for pharmacy professionals when compared to other professionals.

“What we see in other areas is that they have frequent training. If we see prescribers, they have frequent update. Even, if we see the medical team and disease prevention team here, they have regular updating system. Pharmacy professional has no update other than the basic thing from school.” (PS08)

One key informant indicated that the capacity of focal persons at sub-cities is not built on antibiotics and antibiotic resistance and on what should be done at the health facility level to provide the necessary support and follow-up. As a result, they are not providing the necessary support as expected.

Two of the key informants indicated that there was training on medicine use although not completely focused on antibiotics and not provided regularly.

##### 3.3.3.4 Impact of the private sector

The impact of the irrational prescribing of antibiotics from the private sector on the prescribing practice of the public sector was mentioned by almost all of the study participants. The prescribers complained that private health facilities put patients on unnecessarily high-level antibiotics, making patient management, as per the new clinical guidelines, difficult at the health centre level.

“The other big issue now is that private health facilities put patients on ceftriaxone from the beginning. Since they put them on high level antibiotics, managing it with a is not easy when the patients come to the health center.” (HO03)

“I don’t think the private sector is aware of the presence of amoxicillin as a medicine. They directly go for Augmentin or cephalexin. I think this is also one of the contributing factors.” (NU03)

Key informants shared the concerns of prescribers on the prescribing of antibiotics in the private sector and its impact on public health facilities.

“Patients mostly come after visiting private health facilities. As a result, they come to us after reaching third generation. When they come to us, we may put them on first line.” (MS03).

In addition to the private health facilities that prescribe antibiotics (private hospitals and clinics), private pharmacies were also mentioned by many prescribers as having an impact on the prescribing of antibiotics at health centres.

“The things that I think is that there are patients who without visiting a healthcare provider directly go to private pharmacy and ask medicines like any shop for salt or sugar. The professionals in the pharmacy are retailers, businessmen. They give the medicine as per the client’s request. Things like this highly contribute for drug resistance.” (HO10)

MS08 reported another private sector influence on the public sector where pharmaceutical companies influence prescribing by bringing their own protocol while promoting their products.

##### 3.3.3.5 Availability of antibiotics

The availability of antibiotics in the facility was the other common factor that affected prescribing practices. Two prescribers reported that the availability of antibiotics in the facility has an effect on their prescribing practices since they prescribe what is made available in the facility.

“When we prescribe, we consider access. While I am working here, professionals consider the available medicines.” (NU08)

“Yes, especially when the medicines that we want to prescribe are out of stock and the patient does not afford buying from private pharmacies, we are forced to change the medicine. It depends on the medicines made available here.” (NU10)

Some prescribers indicated that they prescribe the antibiotic that is required for the patient’s case irrespective of the availability of the antibiotic in the facility so that the patient can buy from private or public community pharmacies if not available in the facility.

“Especially after we started using the guideline, our cases are guideline related and we don’t hesitate to prescribe as per the guideline. Getting the medicine is up to the patient.” (HO07)

Most of the key informants also indicated that the availability of antibiotics affects the prescriber’s decision.

##### 3.3.3.6 Availability of laboratory tests

The availability of laboratory tests was reported as a factor affecting antibiotic prescribing at health centres. There are shortages in the supply of laboratory reagents, and hence, tests that are necessary to confirm the diagnosis are not undertaken as required in the facilities. A prescriber indicated that the unavailability of laboratory tests affects their prescribing practices.

“Laboratory mostly affects us when we try to manage by ourselves. At times, when we have patient that don’t afford undertaking laboratory test outside, we prescribe based on signs and symptom. The supply is not adequate as per the PHCG.” (NU06)

A key informant from a health centre reinforced this idea by explaining that the overuse of antibiotics is due to a lack of laboratory investigation.

“It is due to the lack of laboratory investigation that there is overuse of antibiotics. If laboratory investigation is available, it would have been possible to identify viral infections which is characterized by the presence of neutrophils.” (PS01)

A key informant from the sub-city health office reported that over half of the laboratory investigations required at the health centre level, as per the PHCG, are not available due to a shortage of laboratory reagents and chemicals. This key informant further highlighted that health facilities may stop providing services if the supply problem continues.

HO07 and NU07 reported that patients are sent to other facilities when laboratory tests are not available in the health facility. In relation to ordering culture and sensitivity tests, a key informant complained about the delay in getting back culture results to make the decision.

“We order culture to be undertaken. We send to Pasteur, Arsho, and others. But, getting the result takes long time. The patient may not even come back to you. We commonly face this. Hence, we may not know the outcome.” (NU10)

##### 3.3.3.7 Failure to engage pharmacy professionals

Almost all of the key informants with a pharmacy background complained that there is a problem that pharmacy professionals are not engaged in implementing new initiatives in which pharmacists can play a critical role. One of the key informants said the following:

“Mostly, I don’t think pharmacy professional is seen as a professional by the government. But, everything is with the pharmacy professional. The knowledge, task, and logistics is with this professional. When something comes, they should update pharmacy professionals together with other health professionals. This is because it is the pharmacist that has direct contact with the medicines and the public.” (PS04)

Most of the complaints were raised in relation to the implementation of the PHCG. According to the key informants, pharmacy professionals were not part of the PHCG; although the guidelines have brought many changes in the management of cases, pharmacy professionals must know how to properly evaluate the appropriateness of the prescription and counsel the patient. They also indicated that a similar complaint was made during the introduction of the Integrated Management of Neonatal and Child Illness (IMNCI) Guideline. The issue is that only prescribers (physicians, health officers, and nurses) are trained without including pharmacy professionals in the training.

According to PS08 and PS09, even pharmacy professionals working at the sub-city level, coordinating pharmaceutical supply and service activities, who are expected to support the health centres, are not trained on the PHCG. Some of them are not even made aware of it as a member of the sub-city health office’s management team.

“I myself don’t know the PHCG. I don’t know it as a system” (PS08)

“I don’t know about that. It is by my own effort that I know about the PHCG. I haven’t taken training. I don’t think those at health center have taken training.” (PS09)

The key informants from sub-cities witnessed that pharmacy professionals working at health centres were not trained on the PHCG and that was a challenge for the implementation of the guidelines at the beginning. The key informants from the Health Bureau indicated that the gap in involving pharmacy professionals in the PHCG training was due to the guidance from MOH to train those involved in case management (physicians, health officers, and nurses), and there was no direction to include pharmacy and laboratory professionals in the training. Later on, the gap was identified once the implementation was started, and these professionals were included in the onsite training provided at health centres together with the rest of the healthcare teams.

##### 3.3.3.8 Lack of guidelines

The lack of clinical guidelines that were seriously and uniformly enforced at each level in the past was mentioned as a contributing factor to the overprescribing of antibiotics.

“Second, we have seen that there was gap in providing similar care for similar clients due to lack of guideline.” (MS01)

Patient load

The mismatch between the number of providers and patients that need services at the health centre level was mentioned by both prescribers and key informants as a factor affecting prescribing practice. According to the study participants, with many patients lined up waiting for service, it is difficult for prescribers to properly diagnose cases and prescribe appropriate antibiotics as per clinical guidelines.

“The first thing is that there is mismatch between the number of patients coming to health facilities and the number of professionals that health facilities have.” (MS09)

##### 3.3.3.9 Inadequate support and follow-up

The key informants feel that the support and follow-up from upper to lower levels in relation to promoting the rational use of medicines is inadequate. One of the key informants did not remember any remedial action taken with respect to antibiotics in the form of support and follow-up from the Health Bureau. Another key informant indicated that the only support they received was PHCG implementation.

##### 3.3.3.10 Lack of accountability

Prescribers often fail to provide full information on prescriptions, especially their professional registration number, to ensure accountability.

“As I was saying, while prescribing, the prescriber should be known as per FMHACA’s standard which indicates that the prescriber’s registration number should be written on the prescription. But, it is not there practically. Even, there are prescribers who do not sign on prescriptions.” (MS11)

## 4 Discussion

The antibiotic prescribing decision of prescribers was shown to be influenced by various patient-, prescriber-, and system-related factors. The factors are related to prescribers, patients, the community, and the healthcare system. The claims from interviewed prescribers who are involved in the practice were supported by the key informants who manage and coordinate the healthcare system at different levels.

Having updated knowledge of antibiotics is key for rational prescribing. For various reasons, prescribers do not update themselves in their area of practice, which leads to competency problems. There are also prescribers who do not properly take patient history, which is very important to know what the patient has already taken and for what reason. This has a lot to do with deciding the management protocol for patients, including the selection of the right antibiotic when necessary. For a healthcare provider practicing at a time when antibiotic resistance is widespread, suspecting AMR should always be at the forefront. Failing to consider AMR during every patient encounter could lead to serious clinical and economic consequences.

Patient pressure and high patient load were among the factors influencing the antibiotic prescribing decisions of prescribers. A study conducted in Cameroon (20) in primary healthcare facilities reported similar findings where time constraints, providers’ perception of patients’ expectation for antibiotics, and patient expectation were among the factors that affected the antibiotic prescribing decision of primary healthcare providers. Patient expectation was also reported as a factor affecting antibiotic prescribing in a study conducted at primary healthcare facilities in Australia (12). The implementation of the CBHI scheme has increased the patient load at healthcare facilities. Furthermore, patients with CBHI membership pressurise healthcare providers to prescribe medicines at every visit despite their case as compensation for the premium they contribute to the scheme.

From the health system perspective, there is a lack of updated information on the local antibiotic resistance situation and antibiotic-specific guidelines, which can serve as reference guides for healthcare providers in prescribing antibiotics. The absence of updated resistance information and clinical guidelines that primary healthcare providers should adhere to leads to irrational prescribing of antibiotics, thereby contributing to the development of antibiotic resistance. Almost all of the study participants witnessed a lack of capacity-building training in the area of antibiotics and resistance. None of the prescribers have taken formal training on antibiotics and resistance, at least during the 2 years preceding the study.

Although some of the prescribers reported that they ordered whatever laboratory test was required for the patient irrespective of the test’s availability in the facility, the diagnostic capacity of the facilities was shown to affect the prescribing decision of prescribers. There is a serious problem in the supply of reagents even to undertake the laboratory investigations that the facilities can conduct. In the absence of laboratory results that can support the decision, the potential for irrational prescribing of antibiotics is very high. There is also a shortage of antibiotics at the primary healthcare facilities, which can limit the prescriber’s ability to prescribe the right antibiotic for the patient. The system is also to be blamed for not having a strong follow-up and support system to improve the prescribing practice at service delivery points and not engaging pharmacy professionals to play their role in improving the antibiotic prescribing and use practice. The system also lacks accountability, which can contribute to improving the prescribing quality.

Most of the study participants agree that the irrational antibiotic-prescribing practice of the private sector is impacting the antibiotics-prescribing practice at public health facilities. That is because the private sector prescribes high-level antibiotics at the patient’s first encounter when no antibiotics or low-level antibiotics can appropriately manage the case. Managing such patients when they come to public health facilities is challenging for healthcare providers working in public health facilities where the practice is guided by clinical guidelines such as PHCG. According to the study conducted on the prescribing pattern of private health facilities in Addis Ababa (21), 63.8% of the prescriptions were found to have one or more antibiotics.

The availability of antibiotics without prescription from private pharmacies and drug stores was the other private sector impact on the public sector’s antibiotic prescribing practice. Although none of the antibiotics are part of the over-the-counter (OTC) list of medicines for Ethiopia (22), dispensing antibiotics without a prescription at private pharmacies and drug stores is very common. A study conducted at private pharmacies and drug stores in Addis Ababa (23) indicated that 63.4% of the simulated visits ended up with over-the-counter dispensing of requested antibiotics, with only 36.6% of the simulated antibiotic requests denied on the grounds of not having a prescription. A study conducted at primary healthcare facilities in Cameroon (20) reported that direct patient access to antibiotics (non-prescription use of antibiotics) was one of the factors that affected the antibiotic prescribing decision of primary healthcare providers. These findings are in line with the claim from the study participants that the private sector is impacting the prescribing practice at public health facilities.

### 4.1 Strengths and limitations

The study identified the factors affecting the prescribing of antibiotics from both prescribers’ and healthcare managers’ perspectives through qualitative enquiry. The findings generated through in-depth interviews of prescribers and key informants involved in managing and coordinating the health service are key to taking appropriate measures to improve the antibiotic prescribing practice. However, the study was conducted only in five of the health centres found in the city administration, which might limit the generalisability of the findings to all of the health centres found in the city administration. Although there were few physicians and midwives involved in the prescribing of medicines at the health centres, only health officers and nurses were involved in the qualitative phase of the study. These categories of professionals might have different views on factors influencing the prescribing of antibiotics than the factors included in the study.

## 5 Conclusion and recommendation

The purpose of the study was to identify and describe factors affecting antibiotic prescribing at primary healthcare facilities in Addis Ababa, Ethiopia. The prescribing of antibiotics is influenced by various prescriber-, patient- and system-related factors. As identifying the underlying factors is a critical step toward addressing a specific problem, this study has generated evidence on factors affecting the prescribing of antibiotics at primary healthcare facilities, thereby paving the way for designing and implementing interventions to improve the practice. Evidence-based interventions should be designed and implemented to address the identified unfavourable factors, thereby improving the prescribing of antibiotics at primary healthcare facilities.

## Author contributions

FA: Conceptualization, Data curation, Formal analysis, Investigation, Methodology, Writing – original draft, Funding acquisition, Project administration, Resources, Visualization. GT-T: Conceptualization, Methodology, Supervision, Validation, Visualization, Writing – review & editing. FM: Conceptualization, Validation, Writing – review & editing, Methodology.
